# Small Non-coding RNA Expression Following Respiratory Syncytial Virus or Measles Virus Infection of Neuronal Cells

**DOI:** 10.3389/fmicb.2021.671852

**Published:** 2021-09-03

**Authors:** Abhijeet A. Bakre, Catherine Duffy, Hani’ah Abdullah, S. Louise Cosby, Ralph A. Tripp

**Affiliations:** ^1^Department of Infectious Diseases, University of Georgia, Athens, GA, United States; ^2^Virology Branch, Veterinary Sciences Division, Agri-Food and Biosciences Institute, Belfast, United Kingdom; ^3^Wellcome Wolfson Institute for Experimental Medicine, School of Medicine, Dentistry and Biomedical Sciences, Queen’s University Belfast, Belfast, United Kingdom

**Keywords:** respiratory syncytial virus, measles virus, neuronal cells, microRNAs, piwi-associated RNAs, transfer RNAs

## Abstract

Respiratory syncytial virus (RSV) or measles virus (MeV) infection modifies host responses through small non-coding RNA (sncRNA) expression. We show that RSV or MeV infection of neuronal cells induces sncRNAs including various microRNAs and transfer RNA fragments (tRFs). We show that these tRFs originate from select tRNAs (GCC and CAC for glycine, CTT and AAC for Valine, and CCC and TTT for Lysine). Some of the tRNAs are rarely used by RSV or MeV as indicated by relative synonymous codon usage indices suggesting selective cleavage of the tRNAs occurs in infected neuronal cells. The data implies that differentially expressed sncRNAs may regulate host gene expression *via* multiple mechanisms in neuronal cells.

## Introduction

Small non-coding RNAs (sncRNAs) are <200 nucleotides and include microRNAs (miRs), PIWI-interacting RNAs (piRs), and transfer RNA fragments (tRNA)-derived RNA fragments (tRFs) (Petrov et al., 2017). The expression of sncRNAs differs in their biogenesis and functional activity (Bartel, 2009; Bakre et al., 2019). Most sncRNAs induced by viral infection involve regulatory mechanisms affecting virus replication, persistence, or latency (Grassmann and Jeang, 2008; Roizman et al., 2011), and immune evasion (Cullen, 2013; Bernier and Sagan, 2018). RSV typically infects ciliated respiratory epithelial cells, while MeV may infect dendritic cells and subsequently infect the epithelia for virus release from the respiratory tract (Lay et al., 2016; Lin and Richardson, 2016; Singh et al., 2016; Taniguchi et al., 2019). These viruses cause a spectrum of diseases and may infect a spectrum of people, i.e., the very young to the elderly (Griffin, 2018; Mazur et al., 2018). RSV is a major cause of serious lower respiratory tract infections in childhood (Mirra et al., 2018; Paes, 2018), but also affects the elderly and immune-compromised (Haber, 2018). MeV causes acute viral infection with respiratory involvement leading to symptoms of rash and more serious complications, and in some cases to mortality (Bohmwald et al., 2018; Leung et al., 2018). Both RSV and MeV are paramyxoviruses with non-segmented negative-sense single-stranded RNA genomes (Russell et al., 2018). Unfortunately, there are no approved RSV vaccines and anti-viral treatments are limited. Although there is an effective MeV vaccine, incidences of MeV infection are increasing worldwide due to vaccine complacency.

Respiratory syncytial virus and MeV can infect neuronal cells, and MeV may cause persistent infection (Rota et al., 2017; Griffin et al., 2018). Compromised immunity may facilitate neuronal infection (Rima and Duprex, 2005; Omar et al., 2017; Ganesan et al., 2018; Ferren et al., 2019). MeV infection of neuronal cells may lead to subacute sclerosing panencephalitis (SSPE), and measles inclusion body encephalitis (MIBE) in immunocompromised individuals. In MIBE, MeV replicates in the central nervous system usually because of an inadequate immune response (McQuaid et al., 1998; Hardie et al., 2013; Rota et al., 2017). RSV infection in mice has been associated with exaggerated neurogenic inflammation in the airways, and studies have shown that the neuropeptide, substance P (SP) is a mediator of neurogenic inflammation (King et al., 2001; Tan et al., 2008).

Expression of the RSV G protein is associated with increased pulmonary expression of SP, and lung neurons express CX3CR1 (Tripp et al., 2000, 2002, 2003; Haynes et al., 2003). CX3CR1 is the receptor for fractalkine (aka neurotactin) and CX3CR1 has been shown to bind RSV G protein (Tripp et al., 2001; Harcourt et al., 2004; Choi et al., 2012; Bakre et al., 2017; Bergeron et al., 2021).

Studies in mice suggest that RSV may cause lasting infection (Dakhama et al., 1997; Li et al., 2006). RSV has been shown to infect mouse primary cortical neuronal cells as shown by co-localization of the RSV N protein and neuronal markers (Li et al., 2006). These findings support how RSV may cause neurological irregularities in patients (Eisenhut, 2006; Li et al., 2006; Morichi et al., 2017). Several studies have described an association between lower respiratory tract RSV infection in infancy and the subsequent development of persistent wheezing in children (Zhou N. et al., 2017; Zhong et al., 2018). In mice, RSV induces long-term airway disease characterized by chronic airway inflammation and airway hyperreactivity (Long et al., 2016; Zhou N. et al., 2017), and RSV antigens have been detected in the lungs >100 days (Schwarze et al., 2004). However, RSV persistence does not appear in the bronchial epithelium (the primary site of viral replication) but in deeper lung structures (Long et al., 2016; Zhou N. et al., 2017).

Respiratory syncytial virus and MeV infection modulate sncRNA expression inducing mRNA degradation or translation inhibition and have a role in determining the level of protein expression of host cells (Munday et al., 2012). It has been shown that tRNAs are cleaved during cellular stress, and that cleavage results in tRFs that contain the 5′ end (5′tRFs) or the 3′ end (3′tRFs) of the parent tRNA molecule (Sofos et al., 2015; Advani and Ivanov, 2019). Although the specific activity of tRFs is not well-understood, tRFs can behave as small interfering RNA leading to the degradation of transcripts (Ivanov et al., 2011) which can regulate ribosomal loading and protein chain elongation (Sobala and Hutvagner, 2013).

Accumulating data have shown that both coding and non-coding transcriptomes are modified during RSV infection. We have shown that RSV infection of human A549 lung cells modifies sncRNA expression and that the RSV G protein and NS1/NS2 proteins modulate miR expression (Bakre et al., 2012, 2015, 2017). Other studies have shown that the pattern of miR expression is modified following RSV infection of nasal epithelial cells (Inchley et al., 2015; Hasegawa et al., 2018), and PBMCs from children (Wang et al., 2017). Deregulated miR expression occurs in MeV-infected human neuroblastoma cells and PBMCs (Inchley et al., 2015; Yis et al., 2015; Naaman et al., 2017; Hasegawa et al., 2018), as well as in PBMCs from RSV-infected children (Wang et al., 2017). It is known that MeV persistence in neuroblastoma cells is assisted by the downregulation of CDK6, a component of cell cycle progression regulated by miRNA-124 (Riddell et al., 2007; Naaman et al., 2017; Griffin et al., 2018).

In this study, we examined sncRNA responses in neuronal SHSY5Y (SHS) cells infected with RSV or MeV and show that infection with either of these viruses modifies miRs and tRF expression in a temporal and virus-specific manner. Anti-microbial peptides (AMPs) are generated in the response to pathogens, and arginine and glycine are especially abundant in AMPs (Mishra and Wang, 2012). In this study, we show that RSV or MeV infection modifies the extent of transfer RNA fragments (tRFs), and the finding of decreased full-length tRNAs after infection indicates decreased translation rates possibly indicative of a lessened anti-viral state. Together, these findings strengthen the notion that sncRNAs have a key role in regulating the host cell response to infection and viral replication.

## Materials and Methods

### Cells and Viruses

Human SHSY5Y (SHS) neuroblastoma cells (ATCC CRL 2266) were grown in DMEM (Sigma, St. Louis, MO, United States) containing 10% FBS (Atlanta Biologicals, Atlanta, GA, United States). SHS cells were maintained at 37°C, 5% CO_2_. RSV strain A2 (ATCC CCL 81) was propagated in Vero cells (Oshansky et al., 2009; Dahiya and Atreya, 2014). Briefly, 80% confluent Vero cells were infected (MOI = 1.0) for 1 h at 37°C, 5% CO_2_. After infection, 2% FBS was added to the DMEM, and the cells were incubated for 3 days until syncytia were evident. On the day of harvest, the cells were removed and centrifuged at 500 × *g* for 15 min at 4°C. RSV strain A2 was used for the infection in SHS cells and rgRSV224 GFP (Hallak et al., 2000, 2007; Techaarpornkul et al., 2002) (a kind gift from Dr. Michael Teng, University of South Florida) for microscopy studies. The WT strain Dublin-3267 of MeV was grown in Vero cells expressing human CD150 (SLAM), a high-affinity receptor for MeV (Tatsuo et al., 2000).

### RNA Isolation and Library Construction

Total RNA from mock-treated or virus-infected SHS cells was isolated using RNAzol RT (MRC, Cincinnati, OH, United States) per the manufacturer’s instructions and divided into small and large RNA fractions and quantified using a Qubit fluorimeter-based RNA assay (Invitrogen Life Tech, Carlsbad, CA, United States). Illumina next-generation sequencing (NGS) libraries were constructed using a TruSeq^®^ small RNA library (Illumina, San Diego, CA, United States). Briefly, small RNA from mock-treated SHS cells (*n* = 2/group), or RSV- or MeV-infected SHS cells (*n* = 3/group) was isolated at 12 or 24 hpi and ligated with 3′ and 5′ RNA adaptors using T4 RNA ligase 2 (Epicenter Biotechnologies, Madison, WI, United States) at 28°C for 60 min. Ligated RNA was reverse transcribed using proprietary reverse primers and the first-strand cDNA was amplified with a combination of a proprietary forward primer and reverse primers containing unique index bar codes per the manufacturer’s instructions. Amplicons were run on a Tapestation 2200 (Agilent, Santa Clara, CA, United States) high-sensitivity DNA chip to visualize products. Indexed samples were pooled per the manufacturer’s protocol and electrophoresed on a 6% TBE-urea PAGE gel. Bands corresponding to 147 and 157 bp corresponding to adaptor-ligated mature miRNA and other non-coding RNAs were cut out and cDNAs were precipitated. Libraries were validated on a Tapestation 2200 (Agilent) using a high-sensitivity DNA chip. Libraries were denatured for 5 min using 5 M NaOH and loaded onto a MiSeq (Agilent) for sequencing by synthesis. Reads were trimmed to remove adaptor sequences and then analyzed for miRNA differential expression using Chimira (Vitsios and Enright, 2015) and OASIS 2.0 (Rahman et al., 2018a). Read counts were normalized to a total number of reads per sample and then differential expression was calculated using DESeq2 (Love et al., 2014). All reads used for analysis had quality scores (*Q*_ > 30. This represents less than or equal to 1 error per 1,000 nts corresponding to a 99.99% accuracy.

### miR Analysis

The host genes and pathways predicted to be regulated by miRs were determined by DIANA miRPath analysis (Vlachos et al., 2015). miRs were examined using the DIANA-miRPath server and targets extracted from the Tarbase database (Karagkouni et al., 2018). Enriched KEGG pathway analysis was used to determine intersections (Kanehisa et al., 2016, 2017). We applied a *p*-value threshold of ≤0.05 along with a false-discovery rate correction.

### Virus Quantitation

QRT-PCR of RSV M, or MeV M, and N gene primer-probes were examined for each replicate to confirm infection of SHS cells. Sequences of the primers/probes used were RSV M forward 5′- ACACCCAAGGGACCTTCACTAAGA, RSV M reverse 5′-GCCTTGATTTCACAGGGTGTGGTT-3′, RSV M probe/56-FAM/AAG TGC AGT GCT AGC ACA AAT GCC CA/3BHQ_1. For MeV, primer sequences were forward primer MeVEdNPF-5′-CAAGACCCTGAGGGATTCAA-3′ and reverse primer MeVEdNPR-5′-CTCTCGCATCACTTGCTCTG-3. For both viruses, 100 ng of RNA was reverse transcribed using Lunascript RT Supermix (New England Biolabs, Ipswitch, MA, United States) using the following conditions: primer annealing at 25°C for 2 min, reverse transcription at 55°C for 30 min followed by heat-inactivation of reverse transcriptase at 95°C for 1 min. Undiluted cDNA was used for PCR. PCR reactions contained cDNA, 0.4 μM of forward and reverse oligomers, 0.8 μM of the probe, 2X Luna Universal probe master mix (New England Biolabs) and were amplified as follows: initial denaturation at 95°C for 60 s, 40 cycles of denaturation at 95°C for 10 s, and combined annealing and extension at 60°C for 30 s. For MeV NP amplification, PCR reactions contained cDNA, 10 μM of forward and reverse primers, Q5 2X High-Fidelity master mix (New England Biolabs), and was amplified using the conditions: initial denaturation at 98°C for 30 s, 25 cycles of 98°C for 10 s, annealing at 65°C for 30 s and extension at 72°C for 2 min, and a final extension at 72°C for 2 min. MeV NP amplicons were resolved on a 1% agarose gel in low osmolality buffer at 5 V/cm and imaged using a Protein Simple imager (Invitrogen).

## Results

### sncRNAs and Virus-Infected SHS Cells

Previous studies from our laboratory have shown that RSV infection of A549 cells modifies miR expression (Bakre et al., 2012, 2015, 2017) however, information regarding miR expression in RSV- or MeV-infected neuronal SHS cells is inadequate (Naaman et al., 2017; Cakmak Genc et al., 2018; Haralambieva et al., 2018). Neuronal cells are not the primary cell type infected by RSV, but they can be infected and may act as a reservoir of infection. Mechanisms that contribute to this are poorly understood. Thus, we examined SHS cells that were RSV- or MeV-infected (MOI = 1.0), or mock-treated and sncRNA expression determined at 12 and 24 hpi by Illumina NGS. We confirmed RSV infection of SHS neuronal cells using RSV-GFP. SHS cells were infected with RSV-GFP at a MOI = 0.1 (Supplementary Figure 1). At 48 h and 72 hpi, virus replication was evident using combined dark field and fluorescence microscopy (UV Eclipse Ti2000-U microscope, Nikon).

**FIGURE 1:**
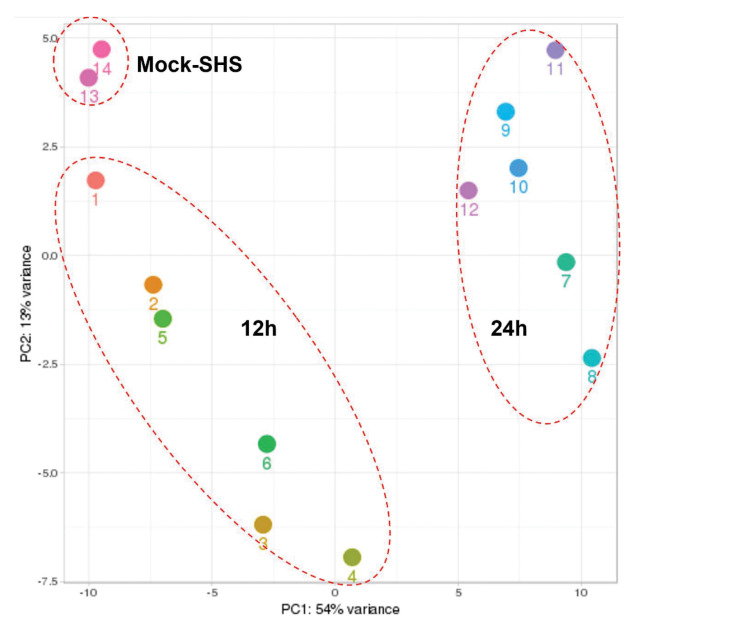
Hierarchical clustering of miR expression. Analysis of overall read composition following RSV- or MeV-infection of SHS cells. Clustering is indicated by dashed circles. 12 h = 12 hpi; 24 h = 24 hpi. Samples 1–3 are 12 h MeV infection, samples 4–6 are 12 h RSV infection, samples 7–9 are 24 h MeV infection, samples 10–12 are 24 h RSV infected SHS cells and mock infected samples are samples 13 and 14. PCA involved all miR, differential expression profiles, and RSV and MeV time points (12 h vs. 24 h) to determine the derived variables known as principal components (PCs). Principal component 1 (PC1) was separated by time (hpi) while principal component 2 (PC2) was separated by infected vs. non-infected.

It is important to note that production of infectious viral particles is restricted in the neuronal environment and viral spread is mediated by fusogenic events leading to syncytia formation that are common to both MeV and RSV infection (Sato et al., 2018). Total reads expressed in a text-based format (fastq) were used and the corresponding reads passing quality scores (*Q* > 30) are listed in Table 1. Raw fastq reads were mapped to the human genome, and miRs using Chimira (Vitsios and Enright, 2015) and Oasis 2.0 (Rahman et al., 2018a) were normalized to the total read counts and used to calculate differential expression using DESeq 2.0 (Love et al., 2014) relative to mock-treated cells. We used principal component analysis (PCA) to determine sample clustering based on similarity for miR expression profiling. PCA is a method of taking combinations of measured variables and lowering the dimensionality of the data to find the principal source of variance. As the human genome encodes > 2,300 miRs and we examined RSV and MeV and two time points, the dataset had > 9,200 variables. PCA identified two major component dimensions—PC1 consists of the time component in addition to virus and miRNA expression, and PC2 is the infection state. PC1 and PC2 are principal components and organized by the percent of variability they explain. Being derived from combinations of variables, PCs do not have labels/units. Samples 1–3 (12 h MeV), 4–6 (12 h RSV), 7–9 (24 h MeV), and 10–12 (24 h RSV) clustered together and away from mock infected cells (samples 13–14) suggesting that the sncRNA response to MeV or RSV infection is distinct from mock infected cells. In addition, clustering of 12 and 24 h MeV and RSV datasets suggests that infections elicit temporal responses. The PCA results indicate that the time post infection is a major component driving differential miRNA expression as shown on PC1 (x-axis with 54% variance) compared to pathogen (PC2 y-axis 13% variance). This is evident in the clustering among mock-treated vs. virus-infected cells (Figure 1). Similarly, miR expression profiles in RSV- or MeV-infected SHS cells at 12 and 24 hpi revealed distinct clustering. Most RSV samples clustered separately from MeV at each time-point (Figure 1).

**TABLE 1:** Summary of read numbers.

Sample	Original # of reads	% Trimmed reads	% Uniquely mapped reads
12 hpi–RSV-1	5,953	98%	5%
12 hpi–RSV-2	18,599	92%	42%
12 hpi–RSV-3	7,546	94%	23%
12 hpi–MeV-1	101,182	78%	50%
12 hpi–MeV-2	27,880	74%	41%
12 hpi–MeV-3	12,088	41%	11%
24 hpi–RSV-1	46,761	72%	10%
24 hpi–RSV-2	239,242	74%	9%
24 hpi–RSV-3	74,687	74%	12%
24 hpi–MeV-1	27,320	66%	7%
24 hpi–MeV-2	26,797	62%	6%
24 hpi–MeV-3	92,746	66%	9%
SHS-1	57,494	79%	51%
SHS-2	77,356	79%	44%

*Numbers indicate raw read counts, percent of reads with adaptor trimming [59], and those mapping to unique loci on the genome.*

The data were filtered to contain only read numbers > 5 (Table 1), and those having *p*-values ≤0.05, and fold-change ≤1.5 or ≥1.5 were used to help identify downregulated vs. upregulated miRs. RSV or MeV infection was confirmed by qPCR. The chief deregulated miRs expressed in SHS cells following RSV- or MeV-infection are shown in Table 2. Compared to mock-infection, expression of miR-10b-5p, miR-181c-5p, and miR-125b-5p were strikingly downregulated in SHS cells at 12 or 24 hpi following RSV or MeV infection. In contrast, expression of p-miR-330, p-miR-338, p-miR-317, p-miR-106, p-miR-113, p-miR-256, p-miR-232, p-miR-103 and miR-182-5p, miR-4532, miR-1273g-3p, miR-483-3p, miR-3141, miR-1246 miR-3196, miR-4516, and miR-4488 were substantially upregulated in SHS cells.

**TABLE 2:** Normalized read counts between mock, RSV- and MeV-infected samples as analyzed by OASIS 2.0 (Rahman et al., 2018a).

			Mock	12 h		24 h	
Mature	log_2_ fold-change	padj	Mock	RSV	MeV	RSV	MeV
miR-10b-5p	−2	0.00	1,416, 1,636	94, 144, 78	369, 262, 128	165, 248, 158	166, 173, 243
miR-181c-5p	−2	0.03	25, 26	12, 3, 6	6, 10, 12	9, 3, 9	10, 5, 6
miR-125b-5p	−2	0.02	50, 34	12, 12, 10	9, 9, 27	12, 10, 10	7, 21, 12
miR-375	2	0.01	56, 49	82, 225, 273	137, 165, 155	168, 132, 193	128, 163, 136
p-miR-247	2	0.02	128, 147	222, 336, 458	131, 298, 530	560, 688, 694	416, 446, 387
miR-21-5p	2	0.03	11, 11	211, 19, 29	43, 23, 15	32, 65, 27	57, 26, 62
miR-877-5p	2	0.02	1, 3	12, 28, 45	5, 10, 19	5, 6, 8	14, 21, 4
miR-7704	3	0.03	1, 1	0, 1, 6	1, 2, 4	25, 16, 17	14, 10, 18
miR-100-5p	3	0.05	0, 1	35, 1, 3	3, 2, 4	2, 1, 4	10, 10, 3
p-miR-330	3	0.01	1, 2	23, 6, 10	3, 4, 15	17, 13, 6	17, 52, 16
miR-182-5p	3	0.00	3, 3	12, 22, 16	34, 30, 23	18, 37, 24	20, 47, 39
miR-4532	4	0.02	0, 0	0, 0, 0	0, 0, 0	11, 29, 10	7, 26, 10
miR-1273g-3p	5	0.00	0, 0	0, 1, 3	0, 1, 0	15, 20, 18	24, 21, 22
miR-483-3p	4	0.00	0, 0	12, 11, 6	3, 10, 12	3, 4, 9	14, 0, 3
miR-3141	5	0.05	0, 0	0, 0, 0	0, 0, 0	29, 32, 14	61, 47, 17
p-miR-338	5	0.01	0, 0	0, 0, 0	0, 0, 0	23, 32, 10	24, 47, 11
miR-1246	5	0.00	0, 1	0, 3, 3	0, 1, 0	32, 42, 22	44, 110, 32
p-miR-317	6	0.00	0, 0	0, 0, 0	0, 0, 0	42, 44, 36	47, 68, 41
miR-3196	6	0.02	0, 0	0, 0, 0	0, 0, 0	29, 52, 14	54, 68, 24
miR-4516	6	0.01	0, 0	0, 0, 3	0, 0, 0	20, 20, 6	41, 26, 11
p-miR-106	6	0.01	0, 0	0, 0, 0	0, 0, 0	22, 18, 12	27, 52, 7
miR-4488	6	0.00	0, 0	0, 0, 0	0, 0, 0	25, 22, 11	34, 63, 15
p-miR-113	6	0.00	0, 0	0, 0, 0	0, 0, 0	37, 61, 35	44, 68, 50
p-miR-256	6	0.00	0, 0	12, 0, 3	0, 1, 0	12, 11, 10	10, 10, 3
p-miR-232	6	0.00	0, 0	0, 0, 0	0, 0, 0	8, 16, 6	20, 26, 9
p-miR-103	7	0.00	0, 0	0, 1, 0	0, 0, 0	22, 29, 21	17, 5, 34

*Negative log_2_ fold-change values indicate downregulation.*

*“p,” predicted miR; RSV, respiratory syncytial virus; MeV, measles virus; padj, Adjusted *p-*value.*

**FIGURE 2:**
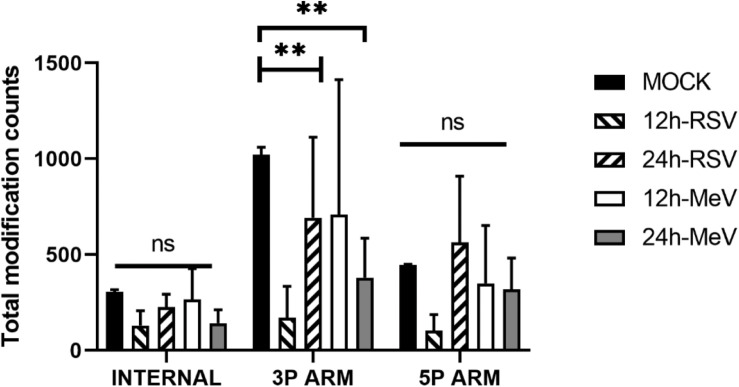
Raw miR reads from RSV- or MeV-infected SHS cells were analyzed for post-transcriptional modifications using Chimira (Vitsios and Enright, 2015). Total counts of internal, 3′ arm and 5′ arm modifications per sample type are shown. Differences in the number of modifications were analyzed by 2-way ANOVA with *post hoc* Dunnet’s test. *p*-values are indicated by ^∗∗^<0.01; ns, not significant. Error bars represent ± SD from three replicate measurements. 3p ARM, 3′ end of miRNA; 5P ARM, 5′ arm of miRNA.

Studies suggest that modifications involving Adenosine to Inosine (A- > I) or Cytosine to Uracil (C- > U) within miRs, N6 methylation, and oxidation affect miR stability, maturation and target specificity (Alarcon et al., 2015; Wang et al., 2015; Correia De Sousa et al., 2019). Post-transcriptional modifications to miRNAs have been shown to alter their binding and function (Pirouz et al., 2019; Briand et al., 2020; Cheray et al., 2020). We therefore examined epitranscriptional modifications of the deregulated miRs using Chimira (Figure 2; Vitsios and Enright, 2015). Most modifications were located on the 3p arms of the deregulated miRs. RSV infection in SHS cells decreased the abundance of 3p modifications at 12 hpi (Figure 2 and Supplementary Table 1).

### RSV or MeV Infection Induces tRNA Fragments

**FIGURE 3:**
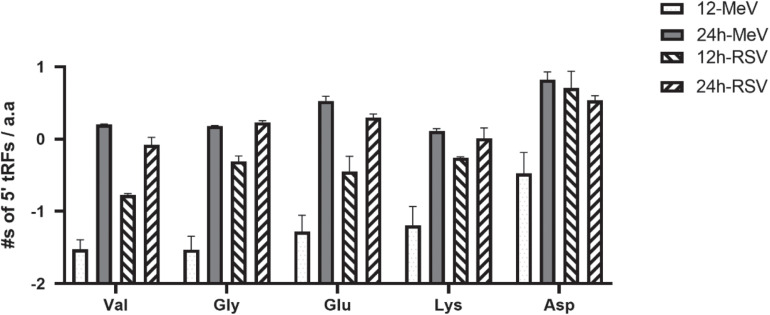
RSV- and MeV -infection influence tRF expression in SHS cells. Total counts for the principal 5′tRFs in MeV- or RSV- infected SHS cells are shown. Error bars represent ± SD from three replicate data. Val, valine; Gly, glycine; Glu, glutamate; Lys, lysine; Asp, aspartate.

**FIGURE 4:**
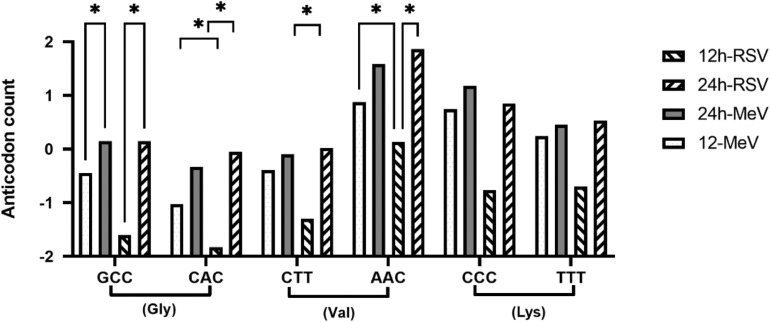
Anti-codons for foremost 5′tRFs during RSV- and MeV-infection. Anti-codon sequence for the top 5′tRFs deregulated in mock, RSV-, or MeV-infected SHS cells. Error bars represent ± SD from three replicate data. Comparisons were done using 2-way ANOVA with a mixed model (to account for outliers) and two replicates for mock followed by *post hoc* Tukey’s correction for multiple comparisons. ^∗^Indicate comparisons with significance *p* ≤ 0.05. Triplets on the x-axis indicate codon sequence.

Cellular stress triggers the degradation of tRNA to tRFs (Shen et al., 2018). tRFs may encompass the 5′, 3′, or internal anti-codon loop and are classified as 5′-, 3-, or class I tRFs (Shen et al., 2018). Functions of tRFs are poorly understood, but tRFs can perform as siRNAs degrading target transcripts (Maute et al., 2013), and regulate ribosomal loading and protein chain elongation (Sobala and Hutvagner, 2013). The biosynthesis of tRFs involves degradation of pre-tRNA molecules *via* the nuclear TRAMP pathway (Anderson, 2005; Maraia and Lamichhane, 2011) or cytosolic degradation of mature tRNAs *via* the rapid tRNA decay pathway. Analysis of the small RNA sequencing dataset in this study showed a differing abundance of tRFs following RSV or MeV infection. Analysis of tRFs showed that among the most abundant were 5′tRFs derived from tRNAs for glycine and valine (Figure 3), suggesting that tRNAs for these two amino acids were targeted during RSV and MeV infection. Levels of 5′tRFs for glycine and valine were substantially increased at 24 hpi following RSV or MeV infection. Frequencies of glycine and valine 5′tRFs increased with time, i.e., from 12 to 24 hpi in RSV- and MeV-infected SHS cells. Analysis of the anti-codon sequence for these tRFs showed that GCC, AAC, and CAC anticodons are enriched in 5′tRFs in SHS cells following RSV or MeV infection (Figure 4). A previous report showed that 5′tRF-Glu-CTC produced during RSV infection of A549 cells interacted with apolipoprotein E receptor 2 (APOER2) mRNA to inhibit translation of the gene, favoring RSV replication (Deng et al., 2015).

**FIGURE 5:**
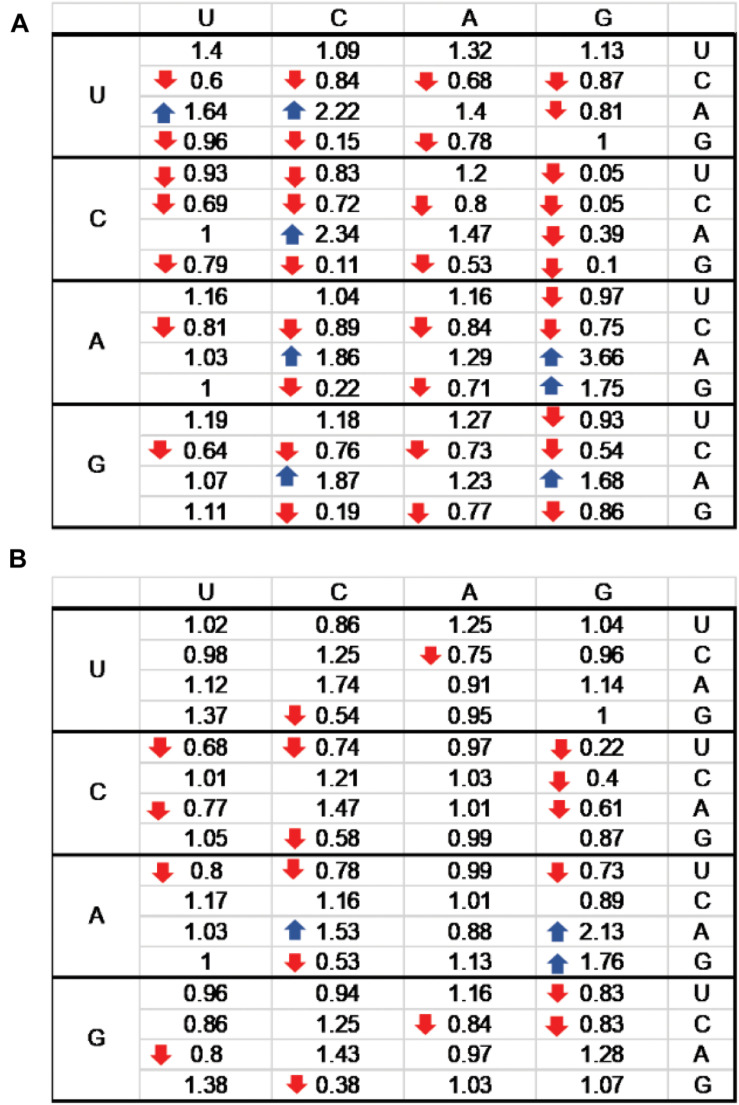
Relative synonymous codon usage (RSCU) index for RSV **(A)** and MeV **(B)** genomes. The standard genetic code table with RSCUs for RSV **(A)** and MeV **(B)** is shown. RSCU indices < 1.0 are indicated by downward arrow icons; RSCU indices > 1.5 are indicated by upward arrows. U, C, A, and G indicate uracil, cytosine, adenosine, and guanosine, respectively.

We postulated that tRFs had a direct effect on translation and compared tRF profiles in RSV- or MeV-infected SHS cells with synonymous codon usage. Our analysis compared how frequently each codon in the RSV or MeV genome is utilized relative to the standard genetic code frequencies that are given as a codon adaptation index (CAI). CAI ranges from 0 to 1.0, with 0 representing least used, and 1.0 representing most common codons. A comparison of RSCU profiles for RSV and MeV genomes using Mega X (Kumar et al., 2018) showed the codon usage to be considerably different than standard human codon usage frequencies (Figures 5A,B). The assessment showed that of the four possible codons that encode glycine (GGU, GGC, GGA, GGG), three codons (GGC, GGU, GGG) are under-utilized for RSV genes, and valine, RSV uses the GUC codon less frequently. For MeV, two of the four codons for glycine (GGU, GGC) and GUA for valine are under-utilized. The consequence of these findings is unknown but suggests that the tRNAs used during RSV or MeV infection are selectively processed to produce these tRNAs potentially for two reasons—to reduce host translation (Kim et al., 2017, 2019; Mesitov et al., 2017), and/or to function as siRNAs and silence host genes (Emara et al., 2010; Wang et al., 2013; Kuscu et al., 2018; Jehn et al., 2020). For valine, and the increased abundance of valine 5′tRFs (AAC and CAC), the corresponding codon usage was not low suggesting that valine tRFs might have functions other than reducing translation rates. RSCU analysis of individual RSV and MeV genes showed that glycine and valine tRNAs were under-utilized following RSV or MeV infection in SHS cells (Table 3). Together these findings suggest that tRNA cleavage is linked with codon usage. The sncRNA results show that the expression profiles for small RNAs differ in kinetics of expression for RSV and MeV infection of SHS cells.

**TABLE 3:** RSCU indices for selected codons for MeV and RSV genes.

Codon	RSV_NS1	RSV_NS2	RSV_NP	RSV_P	RSV_M	RSV_SH	RSV_G	RSV_F	RSV_M2	RSV_L
GUU (V)	0.8	0	1.2	0.9	0.7	0	0	2.0	1.3	1.6
GUG (V)	1.2	0	1.0	2.2	1.4	0	0.5	0.4	0.8	0.6
GGC (G)	1.14	0	0.8	0	0.7	0	0.7	0.7	0	0.5
GGG (G)	0.57	2.0	0.8	1.7	0.7	4.0	0.7	0.3	0.4	0.5

**Codon**	**MeV_N**	**MeV_P**	**MeV_M**	**MeV_F**	**MeV_H**	**MeV_L**				

GUU (V)	2.0	0.9	0.6	0.9	0.6	1.9				
GUG (V)	0.2	2.0	2.3	0.65	1.6	0.7				
GGC (G)	0.7	0.6	1.1	0.8	0.5	1.0				
GGG (G)	0.5	1.3	0.4	1.4	1.8	0.8				

*RSCU scores for individual RSV and MeV genes were determined using MEGA X (Kumar et al., 2018).*

*RSV_NS1, RSV Non-structural protein 1; RSV_NS2, RSV Non-structural gene 2; RSV_NP, RSV Nucleoprotein; RSV_P, RSV Phosphoprotein; RSV_M, RSV Matrix protein; RSV_SH, RSV Small hydrophobic protein; RSV_G, RSV glycoprotein; RSV_F, RSV Fusion protein; RSV_M2, RSV Matrix ORF2 protein; RSV_L, RSV Large polymerase protein; MeV_N, Measles virus Nucleocapsid protein; MeV_P, Measles virus Phosphoprotein; MeV_M, Measles virus Matrix protein; MeV_F, Measles virus fusion glycoprotein; MeV_H, Measles virus hemagglutinin; MeV_L, Measles virus large polymerase.*

## Discussion

In this study, we examined differentially expressed sncRNAs following RSV or MeV infection of SHS cells at 12 and 24 hpi using NGS. NGS showed unique miR expression profiles following RSV or MeV infection (Figure 1 and Table 2). We identified that (1) miR-10b-5p was expressed in mock-treated cells but showed reduced expression in RSV- or MeV-infected cells, (2) p-miR-247 and miR-375 had robust expression in RSV- or MeV-infected cells, (3) miRs had no/low expression in mock-treated cells, but were increased by RSV or MeV infection, and (4) miRs were not expressed in mock-infected cells at 12 hpi but showed robust copy numbers at 24 hpi. These findings show that RSV or MeV infection induces distinct miR expression in SHS cells that is virus-type and time-point specific. While the sequencing data from our study has identified these deregulated miRNAs, the mechanisms contributing to their biogenesis remains unknown and is out of the scope of this manuscript.

Evidence suggests that epitranscriptomics (or post-transcriptional modification of cellular RNAs) especially 3′ end uridylation and adenylation of miRs can alter both miRNA stability and target repertoire (Vitsios and Enright, 2015). We explored the nature of epitranscriptional miR modifications following RSV or MeV infection. The findings suggest that RSV and MeV induce sncRNA modifications affecting target cell specificity. The results showed extensive 3′ modifications (mostly uridylation and adenylation) in the miRs following RSV or MeV infection that may alter host mRNA or miRNA transcript stability. Further studies are needed to elucidate the function of these epitranscriptional modifications on miRNA function.

Post-transcriptional regulatory mechanisms control translation of >60% of the human transcriptome (Friedman et al., 2009). The identification of cleaved tRNAs during RSV or MeV infection of SHS cells is novel. The tRFs identified contained exclusively of 5′tRFs during later stages of RSV or MeV infection, and of these tRNAs, glycine, and valine were the primary targets for 5′tRF formation. Other studies showed that RSV infection can induce angiogenin (ANG)-mediated cleavage of tRNAs to produce tRFs (Deng et al., 2015; Zhou J. et al., 2017). These studies identified a pro-viral role for 5′tRF-GlyCCC, 5′tRF-LysCTT, and 5′tRF-GluCTC by depression of APOER2 mRNA. We postulate that tRNA cleavage to tRFs may have a broader anti-viral role. Degradation of host tRNAs may reduce the anti-viral protein response, and recent findings from proteomic studies in RSV-infected A549 cells have shown that the host nuclear proteins are considerably reduced following RSV infection (Munday et al., 2012, 2015). Similar results have also been shown for MeV, an attribute linked to the N protein (Sato et al., 2007; Okonski and Samuel, 2013). It is important to investigate why RSV or MeV infection induces selective cleavage of glycine and valine tRNAs as glycine, and valine tRNAs are selectively under-utilized by most RSV and MeV genes (Table 3). Transfer RNA fragments operate in mucosal immunity (Chen and Shen, 2021) regulating retrotransposon expression (Schorn et al., 2017) and viral adaptation to the host (Lauring et al., 2012; Pavon-Eternod et al., 2013; Rahman et al., 2018b; Le Nouën et al., 2020; Nunes et al., 2020).

The degradation of tRNAs may be a host stress response to viral infection, however, the increases in tRNA for glycine and valine hint that MeV and RSV may potentially target these tRNAs. The mechanisms that cause are unknown and are difficult to resolve in the context of this study. Currently, we do not understand the mechanisms involved in sncRNA deregulation, nor the functional outcome, thus further studies are necessary. Translation of mRNAs linked to the anti-viral response is key to control of virus infection and clearance. The findings show that RSV or MeV infection induces several classes of sncRNAs that regulate post-transcriptional gene expression and emphasizes the need to better understand the host-virus interface.

## Data Availability Statement

The data presented in the study are deposited in Gene Expression Omnibus (GEO) with accession numbers GSE167724 and GSM5112024–GSM-5112037.

## Author Contributions

AB contributed to conception, execution, and data analysis, and wrote manuscript drafts and revisions. CD contributed to execution. SC and RT contributed to study design, funding, data interpretation, manuscript writing, and review. HA contributed to validating infection using dark field fluorescence microscopy. All authors have seen the draft of the manuscript.

## Conflict of Interest

The authors declare that the research was conducted in the absence of any commercial or financial relationships that could be construed as a potential conflict of interest.

## Publisher’s Note

All claims expressed in this article are solely those of the authors and do not necessarily represent those of their affiliated organizations, or those of the publisher, the editors and the reviewers. Any product that may be evaluated in this article, or claim that may be made by its manufacturer, is not guaranteed or endorsed by the publisher.

## Supplementary Material

Supplementary Figure 1 RSV infects SHS neuronal cells. SHS cells were infected with rgRSV-GFP at MOI = 0.1 for indicated time points. Viral replication was visualized by dark field fluorescence microscopy on a Nikon UV Eclipse TE2000-U microscope at 10X magnification.

Supplementary Table 1 MiRNA modification data patterns in RSV and MeV infected neuronal cells. Table lists samples, microRNAs, type of modifications, modification site, pattern, modification position, internal modification type, internal modification position, repeat modifications and counts of modifications for all deregulated miRNAs identified in the study.
