# Supplementary material for: Small Non-coding RNA Expression Following Respiratory Syncytial Virus or Measles Virus Infection of Neuronal Cells
Source: Front Microbiol. 2021 Sep 3;12:671852. doi: 10.3389/fmicb.2021.671852 (PMC8446675; doi:10.3389/fmicb.2021.671852)
Supplement: Supplementary Figure 1 — RSV infects SHS neuronal cells. SHS cells were infected with rgRSV-GFP at MOI = 0.1 for indicated time points. Viral replication was visualized by dark field fluorescence microscopy on a Nikon UV Eclipse TE2000-U microscope at 10X magnification. [file Data_Sheet_1.docx]

#
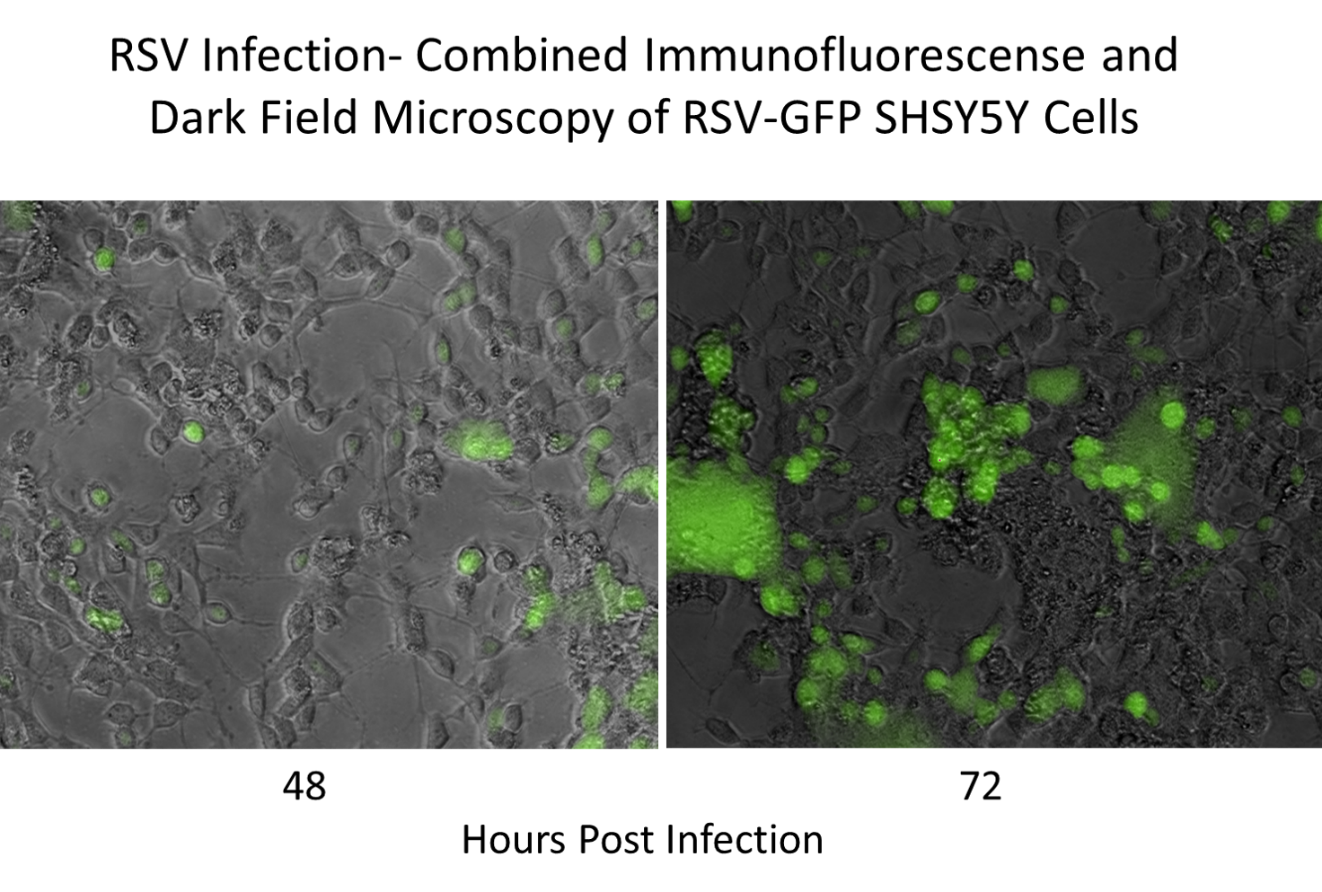
Supplementary Figure 1. RSV infects SHS neuronal cells. SHS cells were infected with rgRSV-GFP at MOI=0.1 for indicated time points. Viral replication was visualized by dark field fluorescence microscopy on a Nikon UV Eclipse TE2000-U microscope at 10X magnification. .
